# Cost-effectiveness of a workplace intervention for sick-listed employees with common mental disorders: design of a randomized controlled trial

**DOI:** 10.1186/1471-2458-8-12

**Published:** 2008-01-14

**Authors:** Sandra H van Oostrom, Johannes R Anema, Berend Terluin, Henrica CW de Vet, Dirk L Knol, Willem van Mechelen

**Affiliations:** 1 Department of Public and Occupational Health, VU University Medical Center, Amsterdam, The Netherlands; 2 EMGO Institute, VU University Medical Center, Amsterdam, the Netherlands; 3 Body@Work, Research Center Physical Activity, Work and Health, TNO-VU, Amsterdam, The Netherlands; 4 Research Center for Insurance medicine AMC-UWV-VU University Medical Center, Amsterdam, The Netherlands; 5 Department of General Practice, VU University Medical Center, Amsterdam, The Netherlands; 6 Department of Clinical Epidemiology and Biostatistics, VU University Medical Center, The Netherlands

## Abstract

**Background:**

Considering the high costs of sick leave and the consequences of sick leave for employees, an early return-to-work of employees with mental disorders is very important. Therefore, a workplace intervention is developed based on a successful return-to-work intervention for employees with low back pain. The objective of this paper is to present the design of a randomized controlled trial evaluating the cost-effectiveness of the workplace intervention compared with usual care for sick-listed employees with common mental disorders.

**Methods:**

The study is designed as a randomized controlled trial with a follow-up of one year. Employees eligible for this study are on sick leave for 2 to 8 weeks with common mental disorders. The workplace intervention will be compared with usual care. The workplace intervention is a stepwise approach that aims to reach consensus about a return-to-work plan by active participation and strong commitment of both the sick-listed employee and the supervisor. Outcomes will be assessed at baseline, 3, 6, 9 and 12 months. The primary outcome of this study is lasting return-to-work, which will be acquired from continuous registration systems of the companies after the follow-up. Secondary outcomes are total number of days of sick leave during the follow-up, severity of common mental disorders, coping style, job content, and attitude, social influence, and self-efficacy determinants. Cost-effectiveness will be evaluated from the societal perspective. A process evaluation will also be conducted.

**Discussion:**

Return-to-work is difficult to discuss in the workplace for sick-listed employees with mental disorders and their supervisors. Therefore, this intervention offers a unique opportunity for the sick-listed employee and the supervisor to discuss barriers for return-to-work. Results of this study will possibly contribute to improvement of disability management for sick-listed employees with common mental disorders. Results will become available in 2009.

**Trial registration:**

ISRCTN92307123

## Background

### CMDs and sick leave

Common mental disorders (CMDs) are common in the community and often affect functioning to such an extent that they are associated with work absenteeism. In many developed countries, 35% to 45% of absenteeism from work is due to mental health problems [1]. Prolonged absence from work often results in a lack of social structure and meaningful activity[2,3] and is associated with a reduced probability of eventual return-to-work (RTW) and an increased probability of subsequent economic and social deprivation [4,5]. In the beginning of the sick leave episode the employee is often missed at work and employers will try to facilitate RTW. However, if sick leave continues, the employee will be replaced and the balance will be regained without the sick-listed employee and therefore RTW will become increasingly difficult to accomplish [6,7]. Thus, work itself can be an important factor in the RTW process[2]. It is also known that work is a significant contributor to the quality of life [8]. Considering the high costs of sick leave and the adverse consequences of sick leave for employees, an early RTW is very important.

### A workplace intervention for CMDs

A recent publication describes the structured development, implementation and planning for the evaluation of a return-to-work (RTW) intervention for sick-listed employees with common mental disorders (CMDs)[9]. The intervention is based on an existing successful RTW intervention for sick-listed employees with low back pain[10,11]. Until now, for mental health problems the focus of interventions is mainly on the reduction of symptoms, while for musculoskeletal disorders the focus has shifted to the prevention of long-term work disability [12]. The main goal of the intervention for employees with low back pain was the facilitation of RTW[10]. Therefore, employees and their supervisors discussed about barriers for RTW and solutions, and they drew up a plan for implementation of solutions which is based on consensus between the employee and the supervisor.

In the development of such an intervention for sick-listed employees with CMDs the steps of Intervention Mapping were followed[13]. In this, important stakeholders in the process of RTW (i.e. employees recently sick-listed with CMDs, supervisors and occupational health professionals) participated in focus group interviews. Topics like, equality and support in discussions about RTW, the role of an RTW coordinator and the suitable moment to apply this workplace intervention for sick-listed employees were discussed. This resulted in a structured return-to-work intervention, specifically tailored to the needs of sick-listed employees with CMDs [9].

### Objective

The objective of this paper is to describe the design of a cost-effectiveness study of the workplace intervention for sick-listed employees with CMDs. This intervention will be compared with usual care.

## Methods

In order to describe the design of this study we followed the CONSORT statement[14,15], a checklist that intends to improve the quality of reports of randomised controlled trials.

### Organisation study

**Figure 1:**
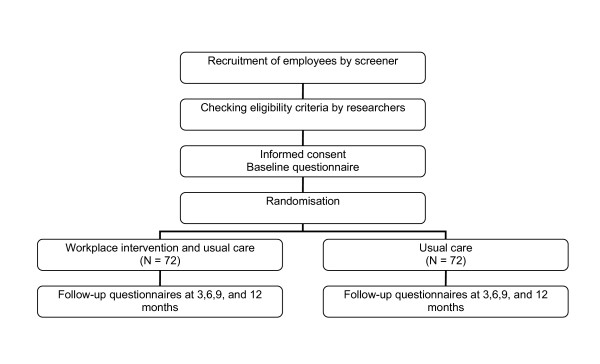
Study design.

The study is designed as a randomised controlled trial with a follow-up of one year. The design is presented in Figure 1. Two occupational health services in the Netherlands collaborate in the study, one occupational health service belongs to the VU University and the VU University Medical Center, and the other is attached to CORUS, a steel industry company.

The Medical Ethics Committee of VU University Medical Center approved the study design, protocols, procedures and informed consent. Participation is voluntary and all participants signed informed consent. Towards the involved stakeholders (employees, supervisors and occupational health professionals) the study is entitled the "ADAPT" study.

### Participants

The source population (n ≈ 20.000) consists of blue and white collar employees working in the University, the University Medical Center and the steel factory. The source population is diverse: the healthcare sector, the industrial sector and administrative sector are all involved in this study. The workplace intervention is assumed to be appropriate for all these sectors.

The employees eligible for this study are on sick leave from regular work for 2 to 8 weeks with CMDs. CMDs encompass both criteria-based psychiatric disorders (mostly depressive and anxiety disorders) and 'subthreshold' disorders (including adjustment disorders)[16]. Employees with CMDs are selected on the basis of elevated distress levels and sick leave. Distress reflects the effort people have to put into coping with stressors in order to maintain their habitual level of psychosocial functioning[17]. Severe distress, however, may lead to a breakdown in coping, resulting in psychosocial disfunctioning (e.g. sick leave). It is generally known that distress and depression coexist with chronic diseases and/or physical symptoms [18-21], therefore a heterogeneous group of participants will be selected.

Employees are in the working age range, 18 to 65 years old. Exclusion of employees occurs in case of 1) a conflict between the employee and the employer with legal involvement; 2) working less than 12 hours a week; 3) pregnancy; 4) sick-listed for more than 8 weeks; 5) another episode of sick leave within one month before the current episode; 6) inability to complete questionnaires written in the Dutch language. After randomisation the occupational physician (OP) is responsible to prevent employees with severe psychiatric disorders (mania, psychosis or suicidal) and employees with a terminal illness from starting the workplace intervention.

### Recruitment of study population

All employees sick-listed for more than one week are selected from the databases of the occupational health services and they are sent a letter from their OP with a screening questionnaire. In the letter, the OP requests the employee to fill in the screening questionnaire and to send it back to the researcher. The screening questionnaire contains three questions based on the distress scale of the Four-Dimensional Symptom Questionnaire (4DSQ) [17,22,23] and a question about whether or not the employee is sick-listed. A screening distress score ≥ 4 corresponds with a 4DSQ distress score ≥ 11, the optimal cut-off for any psychosocial problem [23]. The screening procedure allows the researchers to approach the sick-listed employees in an early stage of sick leave, when other treatments have not yet or only just started, and before the 8 weeks RTW plan is started which is obligated according to the Improved Gatekeeper Act.

Employees who return the questionnaire, meet the distress and sick leave criteria and who indicate 'willing to participate' are contacted by the researchers by telephone. In this contact, the researcher provides additional information about the implications of participation and checks the eligibility of the employee by questions about the six exclusion criteria. If an employee meets all the selection criteria and continues to be willing to participate, written information is provided. The researchers plan a face-to-face appointment with the employee to give consent, fill in the baseline questionnaire and perform the randomisation.

### Usual occupational care in the Netherlands

The Improved Gatekeeper Act regulates, that the responsibility for RTW is given to the employer and employee together. An employer is obliged to start rehabilitation as soon as possible, in order that the employee can resume own work or other adequate work. The employee should accept the work activities the employer provides. Also, the employee has to visit the OP who can provide advice about RTW and who can guide employees on sick leave with CMDs according to the evidence-based guideline of the Dutch Association of Occupational Physicians (NVAB) published in 2000[7,24]. This guideline aims to provide an optimal functioning of the employee with an CMD to prevent long-term sick leave and frequent recurrences. The basic idea of this guideline is that recovery can only appear in interaction with the work-environment and should be based on time-contingency. It starts with the establishment of the diagnosis and a listing of the problems within the private life, the work situation and the health care system. The duration of each stage in ordinary recovery is prescribed in the guideline, hence interventions can be initiated when the OP observes that recovery stagnates.

### Description of the workplace intervention

**Figure 2:**
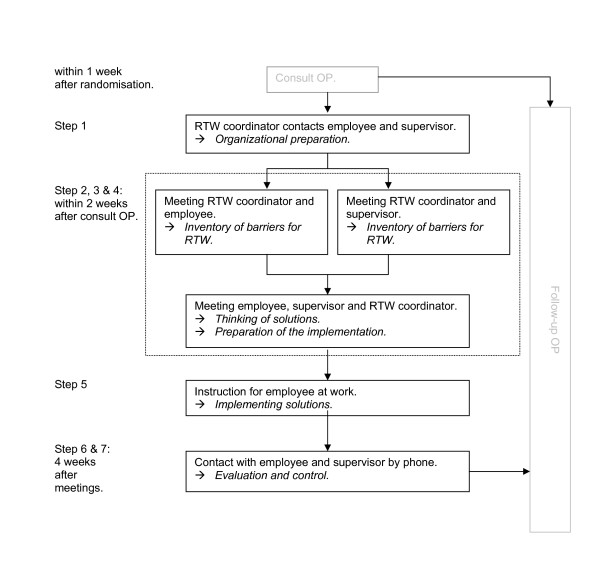
**Content of the workplace intervention**. The steps of the workplace intervention and the stakeholders involved.

The workplace intervention is based on a cost-effective protocol for sick-listed employees with low back pain[10,25]. This protocol is based on principles of 'participatory ergonomics' [26], however applied as a means of tertiary prevention. The process of development of this intervention for employees with CMDs is described elsewhere [9]. The workplace intervention is a stepwise and systematic approach, preceded by a consult with the OP (Figure 2). The objective of the workplace intervention is to reach consensus about an RTW plan by active participation and strong commitment of both the employee and his or her supervisor, guided by an RTW coordinator (in this study a company social worker or a labour expert). The role of the RTW coordinator does not comprise an all knowing expert who advises the employee and the supervisor about the RTW process. The RTW coordinator should provide guidance of the process to reach consensus between the employee and the supervisor about an RTW plan. The employee's and the supervisor's active participation is essential to achieve a sound basis for implementation of the RTW plan.

#### Consult OP

All employees consult the OP before the intervention starts. In this consult the guidelines for usual care are followed. The OP instructs the inventory for stressors to participants as a homework assignment and, if needed, extra consults for stress reduction are planned before the intervention starts. The OP is further responsible to inform the supervisor about the workplace intervention and to ask for his or her participation. To prevent conflicting advise about RTW the OP sends a letter about the workplace intervention and a communication form to the employee's general practitioner. Like in usual care, the OP provides advice about the date of work resumption.

#### Organizational preparation

The RTW coordinator contacts the supervisor to check whether the supervisor is sufficiently informed about the intervention and agrees with it. Next, the RTW coordinator informs who is responsible for work adjustments and what procedures should be followed. The employee and supervisor are contacted by phone to plan the three meetings. If required, a checklist with questions about barriers for RTW are sent to the employee and the supervisor by the RTW coordinator. During the meetings with the employee and the supervisor the RTW coordinator emphasizes that the RTW plan does not implicate that the employee is urged to return to work immediately.

#### Inventory of barriers for RTW

The meeting of the RTW coordinator and the employee starts with a work visit, to observe the employee's workplace. The elements of work content, work environment, communication and collaboration, and work organization are discussed. This provides the RTW coordinator with a complete picture of the work situation.

Then, the RTW coordinator interviews the employee to obtain a description of the main tasks and specific features of these tasks. For each task, barriers for RTW are summarized and are judged based on the frequency and severity of the barrier. Based on this information, barriers are prioritized in order to select the most important.

Subsequently, a meeting between the RTW coordinator and the supervisor intends to select barriers for RTW of the employee from the supervisors' perspective. This procedure is the same as in the interview between the employee and the RTW coordinator. Then, the RTW coordinator summarizes the results of the two interviews and formulates the barriers to be discussed in the next meeting.

#### Thinking of solutions

After the meetings with the employee and the supervisor separately, a meeting takes place with the employee, the supervisor and the RTW coordinator to brainstorm about solutions and to draw up a plan for implementation of solutions. If agreement exist about the barriers to be solved, the RTW coordinator explains the brainstorm procedure. According to the nominal group technique [10] they think of and collect ideas for each solution. All ideas are ordered and judged based on criteria of availability, feasibility and solving capability of the solution and then prioritized. This process is repeated for each barrier. The main goal is to reach consensus between the employee and the supervisor about the most feasible solutions.

#### Preparation of the implementation

Together, the employee, the supervisor and the RTW coordinator formulate a plan for implementation of the solutions. This plan describes who is responsible for the implementation of a solution, how this is planned to occur and when the solution should be implemented. The RTW coordinator writes a report about this plan for implementation of solutions and sends it to the employee, the supervisor and the OP.

#### Implementation of solutions

In the weeks following the meetings, the solutions are implemented. If required, an RTW coordinator plans a meeting in the workplace to instruct and advise the employee at work. For instance, how to deal with a new job performance or with new equipment. At the same time, the supervisor can be informed about how to encourage and guide the employee in his or her (adjusted) work situation.

#### Evaluation by the RTW coordinator and follow-up by the OP

One month after the meetings, the RTW coordinator contacts the employee and the supervisor to inform whether the solutions have been implemented successfully and whether this has contributed to RTW. The RTW coordinator draws up a final report, describing the process and outcome of the implementation and assigns further guidance to the OP.

### Training health care professionals

All OPs involved in this study were trained half a day in the referral of employees to the workplace intervention and the researchers informed them how the workplace intervention is embedded in the guideline for OPs. The referral for the workplace intervention is in line with the guidelines, although explanation about the workplace intervention and standard explanation of the inventory of stressors to the employee is additional. RTW coordinators followed a one-day training course including several role-playings. Each RTW coordinator who guides a first case according to the protocol was contacted by the researchers to facilitate the process. Two follow-up training sessions were conducted during the recruitment period to discuss difficulties and to practise with cases.

### Sample size

Time to lasting RTW is the primary outcome measure for the power calculation. In order to calculate the sample size we assumed that a Hazard Ratio (HR) of 2.0 is the smallest clinical and societal relevant ratio. A HR of 2.0 indicates that employees in the intervention group return to work twice as quickly as employees in the control group. This HR is based on recent studies in occupational health care on RTW of short-term sick-listed employees with low back pain and adjustment disorders[11,25,27,28]. Assuming that a minimum of 2/3 of the participants achieve a full RTW during the follow-up, the calculation showed that a sample size of 98 employees is needed (a power of (1-β =) 0.80 and a two-sided significance level of 0.05 (α)). Since the OPs, because of their role and function in the RTW process, may influence the RTW date, a multilevel analysis on the level of the OP is taken into account. For 13 OPs and an intraclass correlation coefficient (ICC) of 0.05 (clustering in our groups is not presumed to be large) a total of 130 employees are needed. When taking into account a loss to follow-up of 10%, 144 employees have to be enrolled in the study. Although loss to follow-up is taken into account, we do not expect a high loss to follow-up in this study because data about the primary outcome (lasting RTW) will be acquired from the continuous company registration systems after the one-year follow-up.

### Randomisation procedure

An independent statistician prepared the randomisation by using a computer-generated randomisation. To prevent unequal randomisation, employees are pre-stratified by company and whether they are on full or part-time sick leave. Furthermore, block randomisation (with blocks of four) is applied to ensure equal group sizes within each stratum. The researchers prepared sealed envelopes before the start of the study containing either a referral to the workplace intervention group or the usual care group. If the baseline questionnaire is completed, each employee can choose one of the two succeeding envelopes of the correct stratum provided by the researcher. The employee is asked to open the envelope and write down his or her name and the date on the note that contains the randomisation result.

### Blinding

The participants, the occupational health professionals and the researcher are not blinded for the group assignment. It is likely that several participants of one department will participate and therefore knowledge about both groups is provided for the participants. Furthermore, OPs will be visited by both employees in the workplace intervention group and employees in the usual care group. Therefore they cannot be blinded. Since all follow-up questionnaires are sent to the employee by mail, no direct influence by the researchers or occupational health professionals is likely to occur.

The registration of sick leave in the Netherlands is done by companies and managed by the occupational health services. Since these measurements are extracted from computerized databases, bias caused by a lack of blinding is prevented for this outcome. Since the secondary outcomes are all self-reported, blinding is impossible. After randomisation all participants receive a research code consisting of a consecutive number. A research assistant will put all data in the computer by the research code. Therefore, the analysis of the data by the researcher will be blind.

### Co-interventions and compliance

Co-interventions can not be avoided, because less employees will participate when asking to stop with or stay away from other treatments. In both the intervention and control groups co-interventions are assessed in each follow-up measurement. The data about co-interventions on baseline can be used to adjust for co-interventions in the final multivariate analyses if necessary. In the intervention group the compliance to the workplace intervention will be measured by asking employees, supervisors, OPs and RTW coordinators independently about the intervention applied.

### Contamination

As randomisation is performed at the level of the employee, OPs who are trained in the workplace intervention guide participants in both the workplace intervention group and the usual care group. However, the actual intervention will be applied by RTW coordinators. They are asked to avoid application of components of the workplace intervention in case of guidance of employees who are in the usual care group. To prevent contamination and role confusion, RTW coordinators will not apply the workplace intervention for employees from departments they work for regularly.

### Outcomes

This study has a one-year follow-up with measurements scheduled at 3, 6, 9, and 12 months after baseline. After the baseline measurement, all questionnaires are sent to the employees by mail. Data on absenteeism are registered continuously by the companies and will be acquired from the registration systems after the one-year follow-up. These data will be checked with information in the medical file of each employee in the occupational health services. If the data are not consistent, the OP will be asked for clarification. Data about the diagnoses will be obtained from the medical file of each employee. OPs in the Netherlands classify diagnoses according to the Classification of diseases (CAS)[29] which is based on the ICD-10.

#### Effect evaluation

The primary outcome measure in this study is lasting RTW, defined as: duration of sick leave with CMDs in calendar days from the day of randomization until full RTW in own or other work with equal earnings, for at least 4 weeks without (partial or full) recurrence. This means that recurrences of sick leave within 4 weeks of full RTW are considered as belonging to the preceding period of sick leave.

Secondary outcome measures are:

- Total number of days of sick leave. These will be calculated for the entire follow-up period.

- Severity of CMD symptoms. Changes in symptoms of distress, depression, anxiety, and somatization are measured by the Four-Dimensional Symptom Questionnaire (4DSQ). The 4DSQ is a valid self-report questionnaire to measure distress, depression, anxiety and somatization in a working population [17,23].

- Coping style. This outcome is measured with the Ways of Coping Questionnaire (WCQ) [30], which is based on Lazarus' Theory of Stress and Coping[31]. Only two dimensions of the WCQ are included. These dimensions are avoidance and planful problem-solving, to evaluate whether the workplace intervention influences these ways of coping.

- Job content. The Job Content Questionnaire (JCQ)[32] is used to measure job content at baseline and 3 months. Job content data can either be prognostic or provide insight in working mechanism of the workplace intervention.

- Attitude, Social influence and self-Efficacy (ASE) determinants. The ASE-model could provide insight into the working mechanism of the workplace intervention [9]. Questions about attitude, social influence, self-efficacy, and barriers and facilitators were formulated, based on a validated structure of the questions often used in health promotion research[33,34] and are incorporated in the questionnaire at baseline and after 3 months. The questions are measured on bipolar five-point Likert scales.

#### Prognostic measures

Sick leave in the past year, burnout and expectations of the employee about the duration of absence[35] are considered to be potentially prognostic for RTW. Burnout is measured by the Utrecht Burnout Scale-General Survey UBOS[36], the Dutch version of the Maslach Burnout Inventory [37]. In addition, the amount of psychosocial problems may be a prognostic factor for CMDs and sick leave. Psychosocial problems over the last 6 months are measured by the BIOPRO questionnaire [38].

#### Cost-effectiveness evaluation

Cost-effectiveness will be evaluated from the societal perspective. Direct costs of health care usage are measured by the Tic-P questionnaire [39]. The Tic-P is developed for medical costs relevant to the treatment of mental health disorders, such as visits to general practitioner, occupational physician, psychologist, psychiatrist, social worker, admission into a hospital, use of medication etc. Health care costs will be valued according to the prices suggested in the guidelines for economic evaluation in The Netherlands[40]. If cost-guidelines are not available, costs will be estimated using real prices or population-based estimates if available in the literature. Costs of lost productivity caused by (partial) sick leave due to CMDs are calculated from the number of days of sick leave and lost earnings, as provided by the occupational health services. Indirect costs can be calculated using the friction cost approach and the human capital approach, based on income as provided by the employee or as derived from function, age and gender. To compare the results of the cost effectiveness analysis with other conditions, general health status is measured according to the standard Dutch version of the EuroQol EQ-5D[41].

#### Process evaluation

A process evaluation is conducted for the first 35 employees who actually received the workplace intervention. A questionnaire is sent to their supervisor, the OP and the RTW coordinator as well. For employees, the questionnaire is included in the postal questionnaire after 3 months and contains questions about employee satisfaction, the work adaptations chosen, the expected effect of work adaptations, and the compliance with workplace intervention process. Employee satisfaction is measured with the short version of the Patient Satisfaction with Occupational Health Services Questionnaire (PSOHQ)[42]. In addition, the barriers for RTW, the solutions and the RTW plan discussed in the meetings are collected within standardized schemes. All identified barriers for RTW and solutions will be analyzed qualitatively and classified by two researchers independently. The classification will be based on a simplified version of the 'Ergonomic Abstracts' classification scheme [43,44].

### Statistical analyses

All statistical analyses will be performed at employee level, according to the intention-to-treat principle. In order to assess whether protocol deviations have caused bias, the results of the intention-to-treat analyses will be compared to per protocol analyses, excluding those employees who were not treated according to the intervention protocol. Baseline characteristics of employees in the two groups will be compared using descriptive statistics. If necessary, analyses will be adjusted for prognostic dissimilarities.

#### Effect evaluation

Survival analysis will be used to analyse sick leave data with regard to the first period of sick leave. To describe the sick leave duration until lasting RTW in both groups, the Kaplan Meier method will be used. The Cox proportional hazard model will be applied to calculate hazard ratios. If appropriate standard errors will be corrected for clustering. Differences in total days of sick leave during the year of follow-up will be analysed by using the Student's T-test. Longitudinal random coefficient analyses will be used to assess differences in secondary outcome measures. Intraclass correlation coefficients will be calculated.

#### Cost-effectiveness evaluation

Direct, indirect and total costs will be computed for each employee. Bootstrapping will be used for pair-wise comparison of the mean groups to calculate mean differences in direct, indirect and total costs between the two groups of employees. Confidence intervals (95%) will be obtained by bias corrected and accelerated bootstrapping. To assess the cost-effectiveness ratios of the workplace intervention, the difference in mean costs between the groups will be divided by the difference in RTW between the groups. These ratios will be graphically presented in a cost-effectiveness plane. Acceptability curves will also be presented. Similarly, utility assessed with the EuroQol EQ-5D will be used to estimate the incremental costs per QALY gained in a cost-utility analysis.

## Discussion

This study protocol presents a randomised controlled trial to investigate the cost-effectiveness and feasibility of the workplace intervention for sick-listed employees with CMDs. This intervention offers an unique opportunity for the sick-listed employee and his or her supervisor to discuss barriers for RTW related to physical and mental workload. Especially for sick leave due to CMDs, RTW is difficult to discuss in the workplace [45,46]. The screening procedure selects employees who are sick-listed with CMDs. This means that we select not only employees with mental disorders solely, but also employees with a combination of physical and mental complaints. However, application of this intervention and generalizing the results directly to other countries will be difficult because the workplace intervention should always be tailored to the social, political and cultural context [9,47].

### Methodological considerations

A limitation of this study is that it is not possible to blind the employees and health care providers for the intervention allocation, because of the nature of the workplace intervention. The data of the primary outcome will be extracted from databases, therefore no bias will be introduced due to non-blinded employees or OPs for this outcome. A strength of this study is that owing to the screening procedure selection bias is restricted, because the OP has no role in the inclusion of participants. However, selection bias may occur due to self-selection of employees.

There is increasing interest in RTW as an outcome in research. However, little agreement exists about the source of the RTW data. A workers compensation database might underestimate sick-listed days compared with self report by the employee [48,49]. Also, several definitions of RTW are available in the occupational health field [50]. The definition of lasting RTW that we use is the most strictest known and could result in the lowest percentage RTW. For a successful RTW it takes into account not only speed but also durability of RTW. However, in a study that compared several RTW definitions, almost the same magnitude of effect of the interventions was found, regardless of the definition used [51].

Furthermore, we are limited to a one-year follow-up of the sick-listed employees with CMDs. From an economic perspective, a longer follow-up is preferable to investigate intervention effects over several years. Also, productivity loss prior and after the episode of sick leave due to CMDs, is not measured in this study. Even if an employee is not absent from work, CMDs can cause a substantial reduction in productivity [52]. For this study, no information can be generated about differences in productivity losses between the workplace intervention group and the usual care group. Brouwer et al. estimated that productivity loss both prior and after the episode of sick leave can lead to an increase in estimated production losses of about 14% [53].

### Impact of results

The results of this study will possibly contribute to treatment options in occupational health practice, for sick-listed employees with CMDs. In the Netherlands the workplace intervention has already proved to be effective for low back pain. Positive results for sick-listed employees with CMDs could offer extended possibilities for implementation of the workplace intervention in usual care. Occupational health professionals as well as supervisors and employees can possibly benefit from a structured approach to identify and discuss barriers for RTW and the development of an consensus-based RTW plan. Results of this study will become available in 2009.

## Abbreviations

ASE = Attitude-Social influence-self-Efficacy

JCQ = Job Content Questionnaire

CMD = common mental disorder

OP = occupational physician

RTW = return-to-work

UBOS = Utrecht Burnout Scale

WCQ = Ways of Coping Questionnaire

PSOHQ = Patient Satisfaction with Occupational Health Services Questionnaire

4DSQ = Four-Dimensional Symptom Questionnaire

## Competing interests

The author(s) declare that they have no competing interests.

## Authors' contributions

SHO is responsible for the data collection and drafted the manuscript. SHO and JRA developed the study design. JRA was the main applicant and BT and WM were co-applicants on the successful funding proposal. All authors participated in discussing the design of the study and developing the intervention protocols. All authors have read and corrected draft versions of the manuscript and approved the final manuscript.

## Pre-publication history

The pre-publication history for this paper can be accessed here:


